# Individual Differences, Economic Stability, and Fear of Contagion as Risk Factors for PTSD Symptoms in the COVID-19 Emergency

**DOI:** 10.3389/fpsyg.2020.567367

**Published:** 2020-09-08

**Authors:** Adolfo Di Crosta, Rocco Palumbo, Daniela Marchetti, Irene Ceccato, Pasquale La Malva, Roberta Maiella, Mario Cipi, Paolo Roma, Nicola Mammarella, Maria Cristina Verrocchio, Alberto Di Domenico

**Affiliations:** ^1^Department of Neuroscience, Imaging and Clinical Sciences, University “G. d’Annunzio” of Chieti-Pescara, Chieti, Italy; ^2^Department of Psychological, Health and Territorial Sciences, University “G. d’Annunzio” of Chieti-Pescara, Chieti, Italy; ^3^Department of Business Studies, Grenon School of Business, Assumption University, Worcester, MA, United States; ^4^Department of Human Neuroscience, Sapienza University of Rome, Rome, Italy

**Keywords:** COVID-19, PTSD, IES-R, distress, neuroticism, economic stability

## Abstract

On January 30th 2020, the World Health Organization (WHO) declared the COVID-19 pandemic a Public Health Emergency of International Concern (PHEIC). Italy has been one of the most affected countries in the world. To contain further spread of the virus, the Italian government has imposed an unprecedented long-period lockdown for the entire country. This dramatic scenario may have caused a strong psychological distress, with potential negative long-term mental health consequences. The aim of the present study is to report the prevalence of high psychological distress due to the COVID-19 pandemic on the general population, especially considering that this aspect is consistently associated with PTSD symptoms. Furthermore, the present study aims to identify the risk factors for high PTSD symptoms, including individual differences and subjective perception of both economic and psychological aspects. We administered an online survey to 1253 participants during the peak period of the contagion in Italy. A logistic regression on the Impact of Event Scale – Revised (IES-R) scores was used to test the risk factors that predict the possibility to develop PTSD symptoms due to the COVID-19 pandemic. Gender (female), lower perceived economic stability, higher neuroticism, and fear and consequences of contagion were predictors of high PTSD symptomatology. The results, highlighted in the present study, extend our understanding of the COVID-19 pandemic’s impact on the population’s mental health, by identifying individuals at high-risk of developing PTSD. This may help with the implementation of specific protocols to prevent the possibility of developing symptoms of PTSD in target populations.

## Introduction

Coronavirus disease 2019 (COVID-19) arises from SARS-CoV-2, which is an infection that affects the lower respiratory tracts (Ashour et al., 2020; Wölfel et al., 2020). Specifically, COVID-19 symptoms range from asymptomatic infections to mild-severe respiratory symptoms, often accompanied by fever and dry cough, and in some cases, a severe lethal form of pneumonia, acute respiratory distress, and fatality (Rothan and Byrareddy, 2020). It has been estimated that around 20% of COVID-19 patient symptoms will show a severe form of the disease (Zhong et al., 2020). At this time, there is no specific vaccine or treatment for this disease and the elective clinical procedures consist in isolating patients to manage their clinical symptoms. In early December 2019, several cases of this new acute respiratory infection were reported in Wuhan, Hubei Province, China. On January 30th 2020, the World Health Organization (WHO) declared the COVID-19 outbreak as a Public Health Emergency of International Concern (PHEIC) (Mahase, 2020). Although China has been relatively successful in containing its outbreak by reducing new cases of infection by more than 90%, the number of infections spread in other countries, especially Italy, Iran, and United States (Callaway, 2020). Currently, to contain further spread of the virus, governments are implementing unprecedented strict restrictive measures to reduce person-to-person transmission of COVID-19. Consequently, entire nations in different parts of the world have been lockdown, with a full or partial lockdown. The implementation of restrictive measures, such as “social distancing” or “social isolation,” have caused an inevitable readjustment in the daily life of modern societies causing limitations in traveling, social interactions, and work life.

### Psychosocial Impact of COVID-19

Although the psychological impact of COVID-19 pandemic has not yet been well-documented, based on previous experience with coronavirus infections (e.g., MERS-CoV and SARS-CoV), it has been hypothesized that the pandemic is leading to several health problems such as stress, anxiety, depressive symptoms, insomnia, denial, anger, and fear (Torales et al., 2020). In support of this, a study on the psychological impact of the COVID-19, found that more than half of the respondents reported a moderate-to-severe psychological impact, and approximately one-third reported moderate-to-severe anxiety during the initial phase of the outbreak in China (Wang et al., 2020a). Specifically, anxiety levels seem to be related to the fear for contagion of COVID-19, as assessed on an Iranian sample using a new validated self-report questionnaire (Ahorsu et al., 2020). Furthermore, a study on the psychological impact of the lockdown in Italy showed a high increase of distress levels associated to several factors including gender, personality traits, depression and anxiety levels (Mazza et al., 2020).

Beyond the direct effects on mental health, the spread of the pandemic and the consequent restrictive measures are significantly impacting the world economy, resulting in a sharp decline in major financial indices and prompting fear of a global recession (Uddin et al., 2020). Crucially, the ILO Monitor (ILO, 2020), published on April 7th 2020, reports that full or partial lockdown measures, adopted to contain the spread of the virus, are affecting almost 2.7 billion workers globally, which represents around 81% of the entire world’s workforce. Many families are experiencing higher financial distress because of the uncertainty of their incomes. As a result, consumers are reducing spending and are avoiding making new investments (Fernandes, 2020).

Although the psychological and economical long-term effects of the COVID-19 are not yet predictable at this time, it is possible to hypothesize that the COVID-19 emergency is causing drastic changes in the daily life of individuals, causing levels of distress similar to those found in response to traumatic events.

### Factors Predicting PTSD Symptoms

Post-traumatic stress disorder (PTSD) refers to the development of specific negative symptoms after exposure to one or more traumatic events. This symptomatologic presentation may include fear-based re-experiencing, emotional and behavioral changes, dysphoric moods, and negative effects on cognition (American Psychiatric Association, 2013). A self-report questionnaire often used to measure the subjective response to a specific traumatic event, related to the consequent development of PTSD symptoms, is the Impact of Event Scale – Revised (IES-R) (Horowitz et al., 1979). In previous studies, IES-R has been used to evaluate the traumatic impact of past epidemics (SARS, H1N1) during previous cases of lockdown (Hawryluck et al., 2004; Wu et al., 2008, 2009; Wang et al., 2011; Liu et al., 2012). Furthermore, two recent studies used IES-R to measure the psychological distress caused by the COVID-19 pandemic (Wang et al., 2020a,b). These studies highlighted a significant impact of the COVID-19 pandemic in determining high levels of psychological distress, showing, also, differences related to gender with females reporting higher IES-R scores. Several studies have highlighted a similar link between PTSD and gender (Carmassi et al., 2018; Gilmoor et al., 2019). Furthermore, symptoms of PTSD have also been associated to additional variables such as personality traits, socio-economic level, and educational level. Regarding personality traits, the role of neuroticism (alias emotional stability at the opposite end of the continuum) has been widely studied in PTSD. Neuroticism is characterized by aspects of affective negativity (Watson and Tellegen, 1985; McCrae and Costa, 1987) and is constituted by a negative emotional response to frustration, or loss, that often overlaps with specific aspects of arousal symptoms (Yin et al., 2019). Authors have investigated the relationship between neuroticism and stressful events, highlighting the significant correlation between neuroticism, risk of developing PTSD symptoms, and worsening in mental health conditions following a stressful/traumatic event (Holeva and Tarrier, 2001; Engelhard et al., 2003; Frazier et al., 2011). Furthermore, a longitudinal study using IES-R to measure PTSD symptoms, due to the 2004 Tsunami, highlighted that neuroticism was negatively related to PTSD symptoms improvement (6–24 months post-disaster) (Hussain et al., 2013). People who reported high levels of neuroticism tend to react with strong emotions to stressful events. The literature has also highlighted that lower education may be a risk factor in developing PTSD (Carmassi et al., 2018; Kvestad et al., 2019). For instance, education level was associated with the IES-R avoidance score (Wu et al., 2005). Furthermore, a recent meta-analysis highlighted that lower socioeconomic status, lower education level, and gender (female) were predictors of PTSD (Tang et al., 2017). Finally, research highlighted that fear is one of the main factors involved in PTSD (Blechert et al., 2007; Beckers et al., 2013). Notably, during a health crisis the degree of fear can be influenced by the probability of contracting the disease and the consequences derived by it (Yuen et al., 2020).

In the present study we specifically focus on the role of individual differences, perception of economic stability, and psychological factors (including neuroticism and fear for the COVID-19 pandemic) in predicting symptoms of PTSD. We conducted a nationwide survey on a large sample of the Italian population in the period starting from April 1st, 2020 to April 20th, 2020 (the peak of the contagion in Italy, see Supplementary Material). As of May 2020, official data showed that Italy represents one of the most affected countries in the world with approximately 231,000 confirmed cases and more than 33,000 deaths. Furthermore, since March 9th, 2020, the entire country has been experiencing an unprecedented long-term period of lockdown with strict measures including the impossibility for people to leave their home for non-essential reasons, the closure of shops and public spaces, and the ban on gatherings and traveling. This is a crucial aspect considering that a recent review study, on the psychological effects of the lockdown during previous outbreaks, pointed out that individuals experiencing the lockdown showed higher levels of psychological distress compared to their counterparts (Brooks et al., 2020).

## Methods and Measures

### Participants

We recruited 4121 participants using a web-based survey. Economic stability was one of the variables considered for this study. For this reason, we identified individuals who receive a stable income and those who do not receive an income by selecting only unemployed and full-time workers. Other categories such as students, stay-at-home individuals, and retirees were excluded. A total of 1253 (808 female) Italian adults between 18- and 64-years-old (*M* = 39.48, *SD* = 11.94) were included in the present study (see Table 1 for all sample characteristics). The entire survey lasted approximately 15 min. The study was approved by the Board of the Department of Human Neuroscience, Faculty of Medicine and Dentistry, Sapienza University of Rome and all participants provided their consent to participate.

**TABLE 1:** Sample characteristics (Age: *M* = 39.48, *SD* = 11.94) and chi-squared tests.

				**IES-R group**			
		***N***	***%***	**low-PTSDs < *33; N (%)***		**high-PTSDs ≥ *33; N (%)***	
**Total participants**		1253	100.00	807 (64.41)		446 (35.59)	
**Gender**	Female	808	64.50	463 (57.37)	χ^2^ = 35.05**	345 (77.35)	χ^2^ = 266.60**
	Male	445	35.50	344 (42.63)		101 (22.65)	
**Work Status**	Unemployed	328	26.20	203 (25.15)	χ^2^ = 398.43**	125 (28.03)	χ^2^ = 172.03**
	Full-time worker	925	73.80	604 (74.85)		321 (71.97)	
**Education**	High school degree or less	659	52.60	420 (52.04)	χ^2^ = 2.69	239 (53.59)	χ^2^ = 4.59*
	More than high school degree	594	47.40	387 (47.96)		207 (46.41)	
**Home-living condition**	Not alone	1109	88.50	704 (87.24)	χ^2^ = 894.78**	405 (90.81)	χ^2^ = 593.57**
	Alone	114	11.50	103 (12.76)		41 (9.19)	

*p < 0.05, **p < 0.001, Low PTSD symptomatology (low-PTSDs), High-PTSD symptomatology (high-PTSDs).

### Materials and Procedure

The study was administered as a battery of questionnaires using the Qualtrics survey software. The entire survey consisted of two *ad hoc* questionnaires and two standardized measures, described below. Also, a set of socio-demographic questions were presented. Specifically, based on the study hypothesis, we examined gender (male vs. female), work status (full-time worker vs. unemployed), education level (high school degree or less vs. more than high school degree), and home-living condition (not alone vs. alone). Socio-demographic data are shown in Table 1.

#### Fear for COVID-19 (*ad hoc* Questionnaire)

The eight items in the questionnaire were specifically created for the COVID-19 emergency and referred to either self or loved ones’ health. These items are presented in Table 2. Participants answered on a scale from 0 (*not at all*) to 100 (*extremely*). The component structure and reliability of the questionnaire was explored in a larger sample (*n* = 4121), using principal component analysis (PCA) and Cronbach’s alpha. The results from these analyses revealed two factors, with four items per factor. A first factor, “Belief of contagion,” reflects the conviction of being infected, either in the past or in the future. The second factor, “Consequences of contagion,” reflects the possibility of suffering severe consequences due to the contagion (i.e., to be hospitalized or to die). Two scores ranging from 0 to 100 were computed by averaging the items in each scale.

**TABLE 2:** Pattern matrix of the PCA for the Fear for COVID-19 questionnaire.

	**Item**	**Factor loading**	
		**A**	**B**
**(A) Belief of contagion**	I often thought I was infected with the virus	0.734	
	I think I could be infected with the virus in the future	0.802	
	I think that a dear or close person to me could potentially be infected with the virus	0.848	
	I think that a dear or close person to me could potentially be infected with the virus in the future	0.843	
**(B) Consequences of contagion**	I think that a person infected with the virus could recover		0.841
	I think that a person infected with the virus could die		0.800
	I think it is probable that I would recover after being infected with the virus		0.810
	I think that being infected with the virus could be lethal for me		0.721
	Sometimes I have negative thoughts and feelings about the virus*	0.470	0.281

*Item excluded for cross-loadings.

##### PCA of the fear for COVID-19 questionnaire

The factor structure of the questionnaire was evaluated using PCA. An oblique (promax) rotation was used. The scree plot, eigenvalues, and parallel analysis (with 1000 replications) were used to guide the retention of components. The results showed a structure of two moderately correlated factors, *r* = 0.45. The pattern matrix is reported in Table 2. Four items showed satisfactory loadings (i.e., >0.40) on the first factor. These items reflected the conviction to be infected, either in the past or in the future, as well as the beliefs that a loved one has been/will be infected. We labeled this factor “Belief of contagion.” The second factor comprises four items regarding the possibility of suffering severe consequences following contagion (i.e., to be hospitalized, to die), both for her/himself and for a loved one. This factor was labeled “Consequences of contagion.” Only one item showed cross-loadings (i.e., a difference < 0.20 between the loadings on two or more components), which was excluded from the final measure (Howard, 2016). Internal consistency of the final 8-item measure was tested with Cronbach’s alpha. The results showed excellent values for both the Belief of contagion scale, α = 0.82, and Consequences of contagion scale, α = 0.80.

#### Perceived Change in Economic Stability (*ad hoc* Questionnaire)

Two of the questions in the survey dealt with perceived economic stability, either before or during the pandemic. Specifically, the questions were presented as follows: “Before the emergency, I considered my family and I to be economically stable”; and: “During the emergency, I consider my family and I to be economically stable.” Answers were given on a scale from 0 (*not at all*), to 100 (*extremely*). To determine the change in the perceived economic stability, we computed a difference score, labeled as “Economic Stability,” between these two items (before the emergency – during the emergency). Therefore, higher scores on this variable should reflect severe decline in perceived economic stability, while scores approaching zero indicated no changes in personal economic stability. Negative scores, possible but not likely, indicated an improvement in economic stability during the pandemic.

#### The Big Five Inventory 10-Item (BFI-10)

The Big Five Inventory 10-item (BFI-10) is a short scale (Rammstedt and John, 2007) measuring the Big Five personality traits: Agreeableness/Antagonism, Conscientiousness/Lack of direction, Emotional stability/Neuroticism, Extraversion/Introversion, and Openness/Closedness to experience. The BFI-10 has two bidirectional items for each of the Big Five personality factors. Participants are asked to respond to each item indicating whether they agree or disagree with the statement, using a 5-point Likert-type scale, ranging from 1 (*not agree at all*) to 5 (*totally agree*). The scale was developed based on the 44 item Big Five Inventory (Rammstedt, 1997) and designed for contexts in which respondents’ time is severely limited. A previously validated Italian version was used in this study (Guido et al., 2015). In the current study, we focused on Neuroticism (anxiety, angry hostility, depression, self-consciousness, impulsiveness, vulnerability).

#### The Impact of Event Scale – Revised (IES-R)

The Impact of Event Scale – Revised (IES-R) (Christianson and Marren, 2012) assesses the intensity of 22 post-traumatic symptoms pertaining to intrusion, avoidance, and hyper-arousal on a Likert-type scale ranging from 0 (*not at all*) to 4 (*extremely*).

The IES-R was designed and validated providing a specific traumatic event and a specific time frame, as a reference for the subjects. The scale has been found to successfully discriminate between subjects with probable diagnosis of PTSD and subjects with non-probable diagnosis of PTSD. A cut-off score of 33 was found to provide the best accuracy for detection of high levels of PTSD symptoms (Creamer et al., 2003). In this study “COVID-19 epidemic” and “during the emergency” are respectively used for the subjects as a reference of a traumatic event and a specific time frame.

### Statistical Analysis

First, we categorized participants in two groups based on their IES-R total raw score. Specifically, we adopted the optimal cut-off of 33 (Creamer et al., 2003) to distinguish between low PTSD symptoms (low-PTSDs) and high PTSD symptoms (high-PTSDs). We compared the PTSDs groups in terms of individual differences (gender, work status, education, and home-living conditions) performing two-by-two tables chi-squared tests (Campbell, 2007). Based on correlation analysis, we performed a binary logistic regression to predict people’s belonging to low-PTSDs or high-PTSDs group. Specifically, we entered individual differences, perceived change in economic stability, and psychological factors (i.e., neuroticism and fear for COVID-19) as predictors.

## Results

Our aim was to examine the factors leading to high PTSD symptoms related to COVID-19 pandemic. The first striking result was that 35.59% (*N* = 446) of our sample belonged to the high-PTSDs group, reporting a score on IES-R above the cut-off. Furthermore, the low-PTSDs and high-PTSDs groups differed on all individual differences. Specifically, women, full time workers, individuals with high school degree or less, and individuals who did not live alone were more inclined to develop PTSD symptoms compared to men, unemployed individuals, subjects with a higher level of education, and individuals who lived alone respectively. All values are reported in Table 1.

Results of the point-biserial correlations indicated that there was a significant positive association between the IES-R group and “belief of contagion,” “consequences of contagion,” and “economic stability.” Therefore, all these variables could further impact the development of high PTSD symptoms. Furthermore, a significant negative association was found between the “IES-R group” and “Neuroticism,” therefore this personality trait is related to a greater probability of developing PTSD symptoms due to the COVID-19 pandemic. Detailed results of correlations, including means and standard deviations for all variables, are shown in Table 3.

**TABLE 3:** Means, standard deviation, and correlations between variables in the study.

**Variable**	**1**	**2**	**3**	**4**	**5**	**6**	**7**	**8**	**9**	**10**	***M***	***SD***
(1) IES-R group^*a*^	—										—	—
(2) Belief of contagion	0.36**	—									39.60	24.52
(3) Consequences of contagion	0.31**	0.47**	—								47.10	22.70
(4) Neuroticism	−0.34**	−0.23**	−0.26**	—							6.17	2.07
(5) Economic Stability	0.18**	0.12**	0.15**	−0.10**	—						12.02	19.88
(6) Age	−0.02	−0.03	0.06*	0.08**	0.05	—					39.48	11.94
(7) Gender^*a*^	—	−0.11**	−0.12**	0.20**	−0.17**	−0.02	—				—	—
(8) Work Status^*a*^	—	0.09**	−0.04	0.09**	0.01	0.39**	—	—			—	—
(9) Education^*a*^	—	0.08**	−0.09**	0.02	−0.01	−0.04	—	—	—		—	—
(10) Home-living condition^*a*^	—	−0.02*	−0.06**	0.06**	−0.03*	0.03*	—	—	—	—	—	—

^*a*^Point-biserial coefficient, *p < 0.05, **p < 0.001.

Finally, results of the binary logistic regression analysis showed that all entered variables predict the belonging on IES-R groups (see Table 4). Specifically, in the first step gender, work status, education, and home-living conditions were entered. This model explained 6.2% of the variance and only gender resulted as a significant predictor, suggesting that women report higher scores on IES-R. Neuroticism was entered in step 2. The resulting model explained a significant amount of further variance, leading to a total explained variance of 18.2%. Step 3 included perceived change in economic stability, and the effect of this variable was also significant. Specifically, as economic stability goes up, which represents a greater perception of economic instability during the COVID-19 emergency compared to before, the PTSD symptomatology measured by IES-R increases. The total explained variance in step 3 was 20.1%. Finally, in step 4 “belief of contagion” and “consequences of contagion” were entered, and both variables resulted as significant predictors. Hence, increased fear of COVID-19 expressed as the “belief of contagion” and the “consequences of the contagion” also increase the likelihood of being in the high-PTSDs group. The variance explained by the final model was equal to 31.5%.

**TABLE 4:** Summary of Logistic Regression Analysis for variables predicting IES-R group.

**Variable**	**Step 1**					**Step 2**					**Step 3**					**Step 4**				
	***B***	**SE B**	**Wald**	**Exp(B)**	***p***	***B***	**SE B**	**Wald**	**Exp(B)**	***p***	***B***	**SE B**	**Wald**	**Exp(B)**	***p***	***B***	**SE B**	**Wald**	**Exp(B)**	***p***
Gender	0.959	0.136	49.528	2.609	0.000	0.745	0.143	27.240	2.107	0.000	0.652	0.145	20.221	1.919	0.000	0.619	0.153	16.330	1.857	0.000
Work Status	0.064	0.137	0.222	1.067	0.638	−0.048	0.145	0.109	0.953	0.741	−0.020	0.147	0.018	0.981	0.894	0.138	0.156	0.784	1.148	0.376
Education	0.197	0.123	2.560	1.218	0.821	0.122	0.130	0.891	1.130	0.345	0.104	0.131	0.637	1.110	0.425	0.197	0.140	1.986	1.218	0.159
Home-living condition	−0.295	0.201	2.168	0.744	0.141	−0.228	0.209	1.182	0.797	0.277	−0.205	0.210	0.957	0.814	0.328	−0.251	0.220	1.299	0.778	0.254
Neuroticism						−0.346	0.034	106.797	0.707	0.000	−0.343	0.034	103.077	0.709	0.000	−0.286	0.036	63.674	0.751	0.000
Economic stability											0.015	0.003	19.430	1.015	0.000	0.012	0.004	10.922	1.012	0.001
Belief of contagion																0.026	0.003	59.528	1.026	0.000
Consequences of contagion																0.013	0.003	13.931	1.013	0.000
χ*^2^*	57.766					178.306					198.352					325.967				
*Df*	4					5					6					8				
*P*	0.000					0.000					0.000					0.000				
*Nagelkerke R^2^*	0.062					0.182					0.201					0.315				

## Discussion

The COVID-19 epidemic has caused a largescale lockdown worldwide. This pandemic is already showing a high negative impact on physical and mental health. Consequences at the socio-economic level will also be significant which, in turn, will possibly negatively affect mental and emotional stability amongst all individuals.

Little is known about the long-term psychological impact of this pandemic which is characterized by the implementation of public health measures of immense unprecedented magnitude. It appears reasonable to expect an increase of acute stress disorders, PTSDs, emotional, sleep, and depressive disorders because of the emerging effect of several factors, such as the fear of being personally infected or that someone close could be infected (Mucci et al., 2020), and the experience of very negative economic consequences (Marazziti and Stahl, 2020). Furthermore, the impact of all these factors may occur in relation to individual differences. Several studies have been conducted in China; the first country affected by the COVID-19 epidemic. A longitudinal study conducted on 1738 respondents reported the average mean IES-R scores of respondents was above the cut-off score, suggesting a substantial presence of PTSD symptoms among the population (Wang et al., 2020b). Moreover, comparing two-time responses, they found that a prolonged lockdown had an incremental psychological impact on mental health, especially among younger respondents.

Drawing from these findings and considerations, the current study has investigated multiple factors that would influence the psychological impact of COVID-19 among the Italian general population. Our hypothesis about the relation among individual factors, economic stability, and fear of contagion as risk factors for PSTD symptoms related to COVID-19 was supported.

The main striking result of the present study is that, during the peak of the COVID-19 epidemic, more than one-third of the respondents (35.59%; *N* = 446) reported high PTSD symptoms. The rate of individuals with PTSD symptomatology on the Italian population was two times the rate shown in Spain (González-Sanguino et al., 2020). We may hypothesize that higher rates of contagion registered in Italy, at the time of data collection, have caused higher psychological distress in the Italian population.

Also, our results are in line with literature which recognizes the female gender as a risk factor for PTSD symptoms (Christiansen and Elklit, 2008; Ditlevsen and Elklit, 2012). A study reported that women are 6.35 times more likely to have PTSD than men (Pyari et al., 2012). Biological factors are expected to play a role in these differences. For example, women are reported to be more sensitive to stress hormones and threats, less likely to use adaptive coping strategies, and more likely to provide negative appraisal to emergency situations than men (Zhou et al., 2013; Tang et al., 2017). It has also been reported that women tend to assume more caregiving responsibilities. Having to balance work and/or household tasks makes them a group at risk in highly demanding situations (González-Sanguino et al., 2020).

It has also been showed that higher PTSD rates were reported among people with a lower education compared to those with a higher education. Despite conflicting results about the potential relationship between education level and PTSD (Perrin et al., 2007), the strongest evidence seems to suggest that lower levels of education were associated with a higher risk for PTSD (Carmassi et al., 2018; Kvestad et al., 2019) in previous epidemics as well (Wu et al., 2005). As recently highlighted, individuals with a higher level of education and socio-economic status might use better coping strategies because of greater social and economic resources, and ultimately be less impacted by environmental disaster, which in turn reduces the prevalence of PTSD (Tang et al., 2017).

The findings regarding the role of individual factors increasing the risk for PTSD symptomatology, support the consideration that women with a lower educational level, not employed, with higher levels of neuroticism are more at risk to develop emergency trauma-related PTSD symptomatology. It is well known that individuals with higher levels of neuroticism tend to respond with strong emotions to stressful events, experience anxious and depressive affects, tend to appraise events more negatively, and have more difficulty in coping with stressful situations (Suls and Martin, 2005). Each of these factors have been previously considered to propose neuroticism as a risk factor for PTSD in several potential traumatic experiences such as earthquakes, terrorism, and domestic accidents (Breslau and Schultz, 2013; Stevanoviæ et al., 2016; Yin et al., 2019).

Following our regression results, all considered factors, excluding age, work status, education, and the living situation variables, appeared to be important factors in determining high PTSD symptoms due to COVID-19. Specifically, the contributing factors to worsening psychological impact of COVID-19 were gender, neuroticism trait, fear of contagion, and reduced economic stability. A similar study conducted at the time of SARS on 195 adult patients in Hong Kong found higher scores on the avoidance dimension of the IES-R among women (Wu et al., 2005). This evidence was also found among the Spanish population in relation to COVID-19 (González-Sanguino et al., 2020). Considering that PTSD is a fear-based disorder, belief of contagion and consequences of contagion were predictors of PTSD symptoms in Italian adults. Not surprisingly, neuroticism shows a consistent association with higher post-traumatic stress symptoms (Holeva and Tarrier, 2001), and the present study contributes to this knowledge extending the evidence on a pandemic scenario.

The results of the present cross-sectional survey provide relevant data about the post-traumatic psychological distress of COVID-19 in Italy, suggesting the need for greater psychological support in general and especially for high-risk groups. In addition to psychological support, cognitive behavioral therapy (CBT) and eye movement desensitization reprocessing (EMDR) may provide positive effects on core PTSD symptoms. EMDR treatment (Lang, 1977; Bower, 1981) seems to obtain greater results (Moghadam et al., 2020). Reprocessing of eye movement desensitization leads people to overcome feelings of guilt, anxiety, and fear that are typical symptoms deriving from traumatic experiences in general. Since fear of contagion of inappropriate magnitude may result in PTSD (Rau et al., 2005) CBT may help to reduce the level of fear about the dangerousness of COVID-19 and to encourage adaptive emotional responses (Taylor et al., 2020).

Furthermore, the practice of mindfulness is widely used in women (Katz and Toner, 2013; Rojiani et al., 2017) in order to restore a sense of awareness of one’s own experience. The mindfulness based stress reduction (MBSR) technique allows to increase the awareness of responses at a sensorial, affective, and cognitive level. Mindfulness does not require direct exposure of the traumatic event as in most therapeutic strategies targeting PTSD but focuses on the here and now of the subject’s experience (Dutton et al., 2013). Assimilated mindfulness skills can reduce avoidant behaviors related to PTSD by promoting self-management (Gregg et al., 2007) and improving self-compassion (Shapiro et al., 2005; Thompson and Waltz, 2008).

Our results may be helpful to mental health professionals to recognize individuals who are at a higher risk and most in need of interventions, in order to prevent a possible rise of high post-traumatic stress for future infectious disease outbreaks.

Some caveats of the current study need to be acknowledged. First, the data were collected through an online survey, and this may result in participants’ self-selection; hence, we cannot exclude a systematic sampling bias. Second, we used a self-reported questionnaire to investigate PTSD symptoms, however, this administration format may have some biases. The IES-R is a widely used screening tool, scores should not be confused with a diagnosis, which can be obtained only by mental health professionals. Also, the study was conducted during the initial stage of the COVID-19 outbreak; hence, it is possible that we underestimated the actual occurrence of traumatic stress in the population, as delayed onset of PTSD symptoms is conceivable. Third, our study allowed discriminating between people at risk and not at risk for high PTSD symptoms during the COVID-19 pandemic, yet the use of a cross-sectional study design prevented to directly examine causal effects.

Notwithstanding these limitations, this study is a first attempt to elucidate the occurrence of PTSD symptoms in relation to COVID-19 pandemic in the Italian population. Current results extend our knowledge of the links between individual and psychological factors and distress, with potential implication for the general populations’ mental health.

## Conclusion

Our results showed that the COVID-19 pandemic has already had a great psychological impact on the Italian population. Crucially, in the present study more than one-third of the respondents reported PTSD symptoms during the peak of the COVID-19 pandemic. Moreover, it has been highlighted that several individual, economic, and psychological factors play a role in the development of higher levels of PTSD symptomatology. Taken together, these results can provide a benchmark for future studies that aim to focus on the long-term effects of the COVID-19 pandemic. Furthermore, these data can be fundamental in identifying high-risk individuals to reduce the probability of developing PTSD. However, the most important aspect showed in the present study is the need to improve mental healthcare in the immediate future. Therefore, the National Health System and politicians must move in this direction to improve treatment for mental health problems and financial assistance. More professionals (i.e., psychologists, psychiatrists, nurses) should be hired in hospitals and clinics to cope with this emergency in the short and long term (Mucci et al., 2020). In this context, government institutions are called upon to make an effort to provide immediate and long-term financial support in order to fight the war against COVID-19 and try to limit as much as possible the physical, mental, and economic burden.

## Data Availability Statement

The raw data supporting the conclusions of this article will be made available by the authors, without undue reservation.

## Ethics Statement

The studies involving human participants were reviewed and approved by the Board of the Department of Human Neuroscience, Faculty of Medicine and Dentistry, Sapienza University of Rome. The patients/participants provided their written informed consent to participate in this study.

## Author Contributions

ADD, MCV, RP, DM, IC, and NM: conceptualization. ADC, PLM, RM, and MCV: data collection. ADC, IC, RP, DM, and PR: data analyses and interpretation. All authors contributed to writing and review the manuscript.

## Conflict of Interest

The authors declare that the research was conducted in the absence of any commercial or financial relationships that could be construed as a potential conflict of interest.

## References

[B1] Ahorsu D. K. Lin C.-Y. Imani V. Saffari M. Griffiths M. D. Pakpour A. H. (2020). The fear of COVID-19 scale: development and initial validation. *Int. J. Ment. Health Addict.* [Epub ahead of print]. 10.1007/s11469-020-00270-8 32226353 PMC7100496

[B2] American Psychiatric Association (2013). *Diagnostic and Statistical Manual of Mental Disorders*, 5th Edn Philadelphia, PA: American Psychiatric Association.

[B3] Ashour H. M. Elkhatib W. F. Rahman M. M. Elshabrawy H. A. (2020). Insights into the recent 2019 novel coronavirus (SARS-CoV-2) in light of past human coronavirus outbreaks. *Pathogens* 9:186. 10.3390/pathogens9030186 32143502 PMC7157630

[B4] Beckers T. Krypotos A.-M. Boddez Y. Effting M. Kindt M. (2013). What’s wrong with fear conditioning? *Biol. Psychol.* 92 90–96. 10.1016/j.biopsycho.2011.12.015 22223096

[B5] Blechert J. Michael T. Vriends N. Margraf J. Wilhelm F. H. (2007). Fear conditioning in posttraumatic stress disorder: evidence for delayed extinction of autonomic, experiential, and behavioural responses. *Behav. Res. Ther.* 45 2019–2033. 10.1016/j.brat.2007.02.012 17442266

[B6] Bower G. H. (1981). Mood and memory. *Am. Psychol.* 36 129–148. 10.1037/0003-066X.36.2.129 7224324

[B7] Breslau N. Schultz L. (2013). Neuroticism and post-traumatic stress disorder: a prospective investigation. *Psychol. Med.* 43 1697–1702. 10.1017/S0033291712002632 23199934

[B8] Brooks S. K. Webster R. K. Smith L. E. Woodland L. Wessely S. Greenberg N. (2020). The psychological impact of quarantine and how to reduce it: rapid review of the evidence. *Lancet* 395 912–920. 10.1016/S0140-6736(20)30460-8 32112714 PMC7158942

[B9] Callaway E. (2020). Time to use the p-word? Coronavirus enters dangerous new phase. *Nature* 579:12 10.1038/d41586-020-00551-1 33623145

[B10] Campbell I. (2007). Chi-squared and Fisher–Irwin tests of two-by-two tables with small sample recommendations. *Statist. Med.* 26 3661–3675. 10.1002/sim.2832 17315184

[B11] Carmassi C. Gesi C. Corsi M. Cremone I. M. Bertelloni C. A. Massimetti E. (2018). Exploring PTSD in emergency operators of a major University Hospital in Italy: a preliminary report on the role of gender, age, and education. *Ann. Gen. Psychiatry* 17:17. 10.1186/s12991-018-0184-4 29755579 PMC5935923

[B12] Christiansen D. M. Elklit A. (2008). Risk factors predict post-traumatic stress disorder differently in men and women. *Ann. Gen. Psychiatry* 7:24. 10.1186/1744-859X-7-24 19017412 PMC2603007

[B13] Christianson S. Marren J. (2012). The impact of event scale - revised (IES-R). *Medsurg. Nurs.* 21 321–322. 23243796

[B14] Creamer M. Bell R. Failla S. (2003). Psychometric properties of the impact of event scale - revised. *Behav. Res. Ther.* 41 1489–1496. 10.1016/j.brat.2003.07.010 14705607

[B15] Ditlevsen D. N. Elklit A. (2012). Gender, trauma type, and PTSD prevalence: a re-analysis of 18 nordic convenience samples. *Ann. Gen. Psychiatry* 11:26. 10.1186/1744-859X-11-26 23107002 PMC3494556

[B16] Dutton M. A. Bermudez D. Matas A. Majid H. Myers N. L. (2013). Mindfulness-based stress reduction for low-income, predominantly African american women with PTSD and a history of intimate partner violence. *Cogn. Behav. Pract.* 20 23–32. 10.1016/j.cbpra.2011.08.003 24043922 PMC3772725

[B17] Engelhard I. M. van den Hout M. A. Kindt M. (2003). The relationship between neuroticism, pre-traumatic stress, and post-traumatic stress: a prospective study. *Pers. Indiv. Differ.* 35 381–388. 10.1016/S0191-8869(02)00200-3

[B18] Fernandes N. (2020). *Economic Effects of Coronavirus Outbreak (COVID-19) on the World Economy.* Rochester, NY: Social Science Research Network.

[B19] Frazier P. Gavian M. Hirai R. Park C. Tennen H. Tomich P. (2011). Prospective predictors of posttraumatic stress disorder symptoms: direct and mediated relations. *Psychol. Trauma Theory Res. Pract. Policy* 3 27–36. 10.1037/a0019894

[B20] Gilmoor A. R. Adithy A. Regeer B. (2019). The cross-cultural validity of post-traumatic stress disorder and post-traumatic stress symptoms in the indian context: a systematic search and review. *Front. Psychiatry* 10:439. 10.3389/fpsyt.2019.00439 31333512 PMC6620607

[B21] González-Sanguino C. Ausín B. ÁngelCastellanos M. Saiz J. López-Gómez A. Ugidos C. (2020). Mental health consequences during the initial stage of the 2020 Coronavirus Pandemic (COVID-19) in Spain. *Brain Behav. Immun.* 87 172–176. 10.1016/j.bbi.2020.05.040 32405150 PMC7219372

[B22] Gregg J. A. Callaghan G. M. Hayes S. C. Glenn-Lawson J. L. (2007). Improving diabetes self-management through acceptance, mindfulness, and values: a randomized controlled trial. *J. Consult. Clin. Psychol.* 75 336–343. 10.1037/0022-006X.75.2.336 17469891

[B23] Guido G. Peluso A. M. Capestro M. Miglietta M. (2015). An Italian version of the 10-item big five inventory: an application to hedonic and utilitarian shopping values. *Pers. Indiv. Differ.* 76 135–140. 10.1016/j.paid.2014.11.053

[B24] Hawryluck L. Gold W. L. Robinson S. Pogorski S. Galea S. Styra R. (2004). SARS control and psychological effects of quarantine. Toronto, Canada. *Emerg. Infect. Dis.* 10 1206–1212. 10.3201/eid1007.030703 15324539 PMC3323345

[B25] Holeva V. Tarrier N. (2001). Personality and peritraumatic dissociation in the prediction of PTSD in victims of road traffic accidents. *J. Psychosom. Res.* 51 687–692. 10.1016/S0022-3999(01)00256-2 11728510

[B26] Horowitz M. Wilner N. Alvarez W. (1979). Impact of event scale: a measure of subjective stress. *Psychosom. Med.* 41 209–218. 10.1097/00006842-197905000-00004 472086

[B27] Howard G. R. (2016). *We Can’t Teach What We Don’t Know, Third Edition: White Teachers, Multiracial Schools.* New York, NY: Teachers College Press.

[B28] Hussain A. Weisæth L. Heir T. (2013). Posttraumatic stress and symptom improvement in Norwegian tourists exposed to the 2004 tsunami – a longitudinal study. *BMC Psychiatry* 13:232. 10.1186/1471-244X-13-232 24063414 PMC3851444

[B29] ILO (2020). *ILO Monitor: COVID-19 and the World of Work. 2nd Edn.* Available online at: https://www.ilo.org/global/about-the-ilo/WCMS_740877/lang–en/index.htm (accessed 20 April 2020)

[B30] Katz D. Toner B. (2013). A systematic review of gender differences in the effectiveness of mindfulness-based treatments for substance use disorders. *Mindfulness* 4 318–331. 10.1007/s12671-012-0132-3

[B31] Kvestad I. Ranjitkar S. Ulak M. Chandyo R. K. Shrestha M. Shrestha L. (2019). Earthquake exposure and post-traumatic stress among nepalese mothers after the 2015 earthquakes. *Front. Psychol.* 10:734. 10.3389/fpsyg.2019.00734 31001178 PMC6454014

[B32] Lang P. J. (1977). Imagery in therapy: an information processing analysis of fear. *Behav. Ther.* 8 862–886. 10.1016/S0005-7894(77)80157-3 27816081

[B33] Liu X. Kakade M. Fuller C. J. Fan B. Fang Y. Kong J. (2012). Depression after exposure to stressful events: lessons learned from the severe acute respiratory syndrome epidemic. *Comprehen. Psychiatry* 53 15–23. 10.1016/j.comppsych.2011.02.003 21489421 PMC3176950

[B34] Mahase E. (2020). China coronavirus: WHO declares international emergency as death toll exceeds 200. *Br. Med. J.* 368 m408. 10.1136/bmj.m408 32005727

[B35] Marazziti D. Stahl S. M. (2020). The relevance of COVID-19 pandemic to psychiatry. *World Psychiatry* 19 261–261. 10.1002/wps.20764 32394565 PMC7215065

[B36] Mazza C. Ricci E. Biondi S. Colasanti M. Ferracuti S. Napoli C. (2020). A nationwide survey of psychological distress among Italian people during the COVID-19 pandemic: immediate psychological responses and associated factors. *Int. J. Environ. Res. Public Health* 17:3165. 10.3390/ijerph17093165 32370116 PMC7246819

[B37] McCrae R. R. Costa P. T. (1987). Validation of the five-factor model of personality across instruments and observers. *J. Pers. Soc. Psychol.* 52 81–90. 10.1037/0022-3514.52.1.81 3820081

[B38] Moghadam S. Kazemi R. Taklavi S. Naeim M. (2020). Comparing the effectiveness of eye movement desensitization reprocessing and cognitive behavioral therapy in reducing post-traumatic stress disorder. *Health Psychol. Rep.* 7.

[B39] Mucci F. Mucci N. Diolaiuti F. (2020). Lockdown and isolation: psychological aspects of COVID-19 pandemic in the general population. *Clin. Neuropsychiatry* 17 63–64. 10.36131/CN20200205 PMC8629090 34908969

[B40] Perrin M. A. DiGrande L. Wheeler K. Thorpe L. Farfel M. Brackbill R. (2007). Differences in PTSD prevalence and associated risk factors among World Trade Center disaster rescue and recovery workers. *Am. J. Psychiatry* 164 1385–1394. 10.1176/appi.ajp.2007.06101645 17728424

[B41] Pyari T. T. Kutty R. V. Sarma P. S. (2012). Risk factors of post-traumatic stress disorder in tsunami survivors of Kanyakumari District. Tamil Nadu, India. *Indian J. Psychiatry* 54 48–53. 10.4103/0019-5545.94645 22556437 PMC3339219

[B42] Rammstedt B. (1997). *Die deutsche Version des Big Five Inventory (BFI): Übersetzung und Validierung eines Fragebogens zur Erfassung des Fünf-Faktoren-Modells der Persönlichkeit.* Available online at: https://madoc.bib.uni-mannheim.de/54690/ (accessed 28 May 2020).

[B43] Rammstedt B. John O. P. (2007). Measuring personality in one minute or less: a 10-item short version of the Big Five Inventory in English and German. *J. Res. Personality* 41 203–212. 10.1016/j.jrp.2006.02.001

[B44] Rau V. DeCola J. P. Fanselow M. S. (2005). Stress-induced enhancement of fear learning: an animal model of posttraumatic stress disorder. *Neurosci. Biobehav. Rev.* 29 1207–1223. 10.1016/j.neubiorev.2005.04.010 16095698

[B45] Rojiani R. Santoyo J. F. Rahrig H. Roth H. D. Britton W. B. (2017). Women benefit more than men in response to college-based meditation training. *Front. Psychol.* 8:551. 10.3389/fpsyg.2017.00551 28473783 PMC5397480

[B46] Rothan H. A. Byrareddy S. N. (2020). The epidemiology and pathogenesis of coronavirus disease (COVID-19) outbreak. *J. Autoimmun.* 109:102433. 10.1016/j.jaut.2020.102433 32113704 PMC7127067

[B47] Shapiro S. Astin J. Bishop S. Cordova M. (2005). Mindfulness-based stress reduction for health care professionals: results from a randomized trial. *Int. J. Stress Manag.* 12 164–176. 10.1037/1072-5245.12.2.164

[B48] Stevanoviæ A. Franèiškoviæ T. Vermetten E. (2016). Relationship of early-life trauma, war-related trauma, personality traits, and PTSD symptom severity: a retrospective study on female civilian victims of war. *Eur. J. Psychotraumatol.* 7:30964. 10.3402/ejpt.v7.30964 27056034 PMC4824847

[B49] Suls J. Martin R. (2005). The daily life of the garden-variety neurotic: reactivity, stressor exposure, mood spillover, and maladaptive coping. *J. Pers.* 73 1485–1510. 10.1111/j.1467-6494.2005.00356.x 16274443

[B50] Tang B. Deng Q. Glik D. Dong J. Zhang L. (2017). A meta-analysis of risk factors for Post-Traumatic Stress Disorder (PTSD) in adults and children after earthquakes. *Int. J. Environ. Res. Public Health* 14:1537. 10.3390/ijerph14121537 29292778 PMC5750955

[B51] Taylor S. Landry C. A. Paluszek M. M. Fergus T. A. McKay D. Asmundson G. J. G. (2020). COVID stress syndrome: concept, structure, and correlates. *Depression and Anxiety* [Epub ahead of print] 10.1002/da.23071 32627255 PMC7362150

[B52] Thompson B. L. Waltz J. (2008). Self-compassion and PTSD symptom severity. *J. Traum. Stress* 21 556–558. 10.1002/jts.20374 19107727

[B53] Torales J. O’Higgins M. Castaldelli-Maia J. M. Ventriglio A. (2020). The outbreak of COVID-19 coronavirus and its impact on global mental health. *Int. J. Soc. Psychiatry* 66 317–320. 10.1177/0020764020915212 32233719

[B54] Uddin M. Mustafa F. Rizvi T. A. Loney T. Suwaidi H. A. Marzouqi A. A. (2020). SARS-CoV-2/COVID-19: viral genomics, epidemiology, vaccines, and therapeutic interventions. *Viruses* 12:526. 10.20944/preprints202004.0005.v1 32397688 PMC7290442

[B55] Wang C. Pan R. Wan X. Tan Y. Xu L. Ho C. S. (2020a). Immediate psychological responses and associated factors during the initial stage of the 2019 Coronavirus Disease (COVID-19) epidemic among the general population in China. *Int. J. Environ. Res. Public Health* 17:1729. 10.3390/ijerph17051729 32155789 PMC7084952

[B56] Wang C. Pan R. Wan X. Tan Y. Xu L. McIntyre R. S. (2020b). A longitudinal study on the mental health of general population during the COVID-19 epidemic in China. *Brain Behav. Immun.* 87 40–48. 10.1016/j.bbi.2020.04.028 32298802 PMC7153528

[B57] Wang Y. Xu B. Zhao G. Cao R. He X. Fu S. (2011). Is quarantine related to immediate negative psychological consequences during the 2009 H1N1 epidemic? *Gen. Hosp. Psychiatry* 33 75–77. 10.1016/j.genhosppsych.2010.11.001 21353131

[B58] Watson D. Tellegen A. (1985). Toward a consensual structure of mood. *Psychol. Bull.* 98 219–235. 10.1037/0033-2909.98.2.219 3901060

[B59] Wölfel R. Corman V. M. Guggemos W. Seilmaier M. Zange S. Müller M. A. (2020). Virological assessment of hospitalized patients with COVID-2019. *Nature* 581 465–469. 10.1038/s41586-020-2196-x 32235945

[B60] Wu K. K. Chan S. K. Ma T. M. (2005). Posttraumatic stress, anxiety, and depression in survivors of severe acute respiratory syndrome (SARS). *J. Traum. Stress* 18 39–42. 10.1002/jts.20004 16281194 PMC7166878

[B61] Wu P. Fang Y. Guan Z. Fan B. Kong J. Yao Z. (2009). The psychological impact of the SARS epidemic on hospital employees in China: exposure, risk perception, and altruistic acceptance of risk. *Can. J. Psychiatry* 54 302–311. 10.1177/070674370905400504 19497162 PMC3780353

[B62] Wu P. Liu X. Fang Y. Fan B. Fuller C. J. Guan Z. (2008). Alcohol abuse/dependence symptoms among hospital employees exposed to a SARS outbreak. *Alcohol Alcohol* 43 706–712. 10.1093/alcalc/agn073 18790829 PMC2720767

[B63] Yin Q. Wu L. Yu X. Liu W. (2019). Neuroticism predicts a long-term PTSD after earthquake trauma: the moderating effects of personality. *Front. Psychiatry* 10:657. 10.3389/fpsyt.2019.00657 31616324 PMC6763688

[B64] Yuen K. F. Wang X. Ma F. Li K. X. (2020). The psychological causes of panic buying following a health crisis. *Int. J. Environ. Res. Public Health* 17 3513. 10.3390/ijerph17103513 32443427 PMC7277661

[B65] Zhong B.-L. Luo W. Li H.-M. Zhang Q.-Q. Liu X.-G. Li W.-T. (2020). Knowledge, attitudes, and practices towards COVID-19 among Chinese residents during the rapid rise period of the COVID-19 outbreak: a quick online cross-sectional survey. *Int. J. Biol. Sci.* 16 1745–1752. 10.7150/ijbs.45221 32226294 PMC7098034

[B66] Zhou X. Kang L. Sun X. Song H. Mao W. Huang X. (2013). Prevalence and risk factors of post-traumatic stress disorder among adult survivors six months after the Wenchuan earthquake. *Comprehen. Psychiatry* 54 493–499. 10.1016/j.comppsych.2012.12.010 23337407

