# Supplementary material for: Individual Differences, Economic Stability, and Fear of Contagion as Risk Factors for PTSD Symptoms in the COVID-19 Emergency
Source: Front Psychol. 2020 Sep 8;11:567367. doi: 10.3389/fpsyg.2020.567367 (PMC7506146; doi:10.3389/fpsyg.2020.567367)
Supplement: Supplementary file 1 [file Data_Sheet_1.pdf]

Supplementary Figures

**Supplementary Figure 1.** *Cumulative number of cases and deaths in Italy from March 9th to April 20th.*

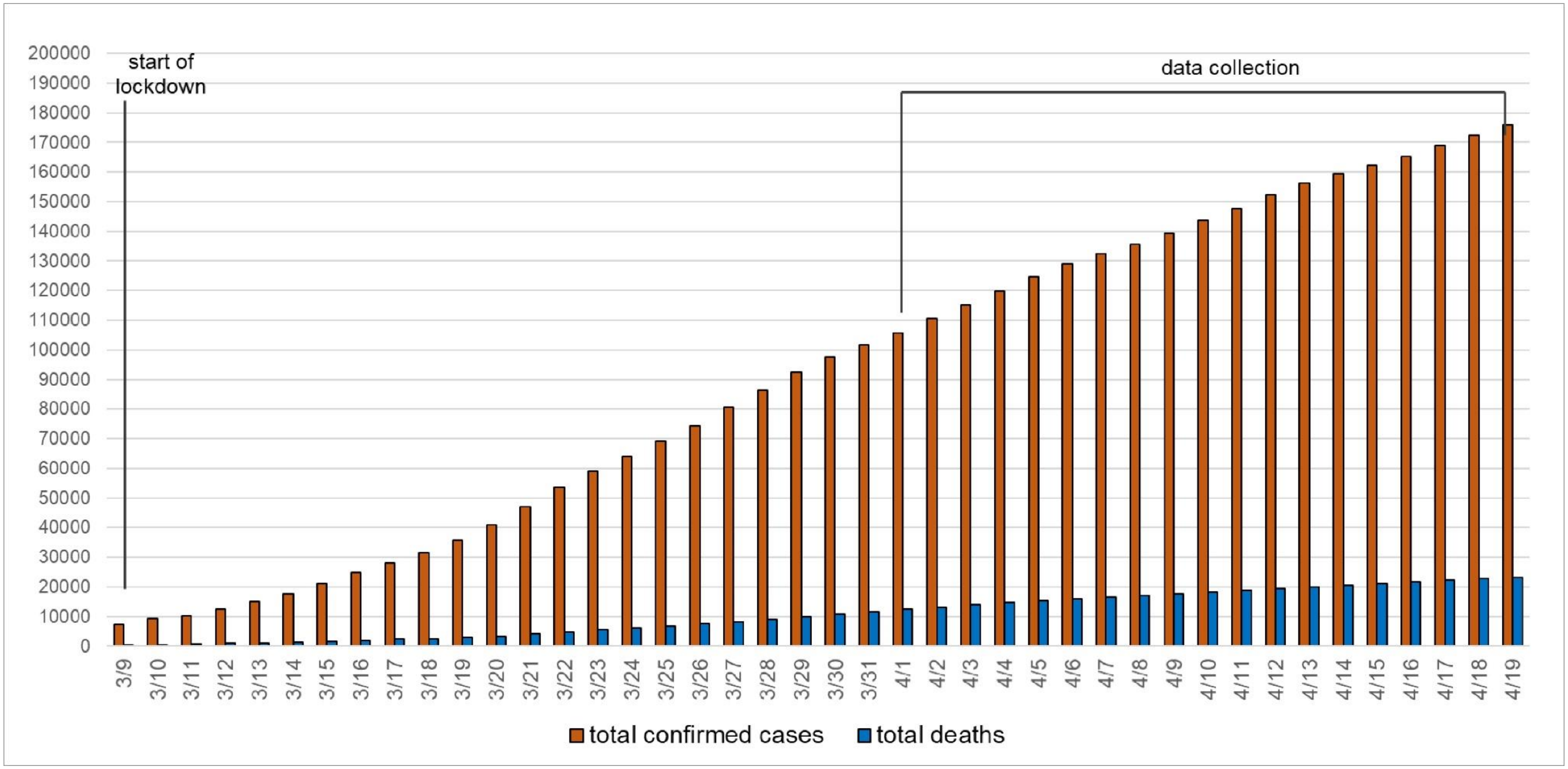


***Note.***

*Source: WHO Coronavirus disease (COVID-2019) situation reports, https://www.who.int/emergencies/diseases/novel-coronavirus-2019/situation-reports*
